# One-Off Irrigation Combined Subsoiling and Nitrogen Management Enhances Wheat Grain Yield by Optimizing Physiological Characteristics in Leaves in Dryland Regions

**DOI:** 10.3390/plants13243526

**Published:** 2024-12-17

**Authors:** Ming Huang, Shuai Zhang, Mengqi Yang, Yuhao Sun, Qinglei Xie, Cuiping Zhao, Kaiming Ren, Kainan Zhao, Yulin Jia, Jun Zhang, Shanwei Wu, Chunxia Li, Hezheng Wang, Guozhan Fu, Muhammad Shaaban, Jinzhi Wu, Youjun Li

**Affiliations:** 1 College of Agriculture, Henan University of Science and Technology, Luoyang 471023, China; huangming_2003@126.com (M.H.); zhangshuai089419@163.com (S.Z.); yangmq200109@163.com (M.Y.); sunyuhao202410@163.com (Y.S.); xieqinglei101@163.com (Q.X.); zhaocuiping883193@163.com (C.Z.); renkaiming1848@163.com (K.R.); zhaokainan6@126.com (K.Z.); jiayulinjyl@163.com (Y.J.); bjzhangjun@126.com (J.Z.); wsw1223@163.com (S.W.); libingxin1997328@126.com (C.L.); wanghz@126.com (H.W.); luoyangfuguozhan@163.com (G.F.); shabanbzu@hotmail.com (M.S.); 2 Crop Research Institute, Shandong Academy of Agricultural Sciences, Jinan 250100, China

**Keywords:** irrigation practice, tillage method, nitrogen management, drought-prone region, winter wheat, grain yield, physiological characteristics

## Abstract

Irrigation practice, tillage method, and nitrogen (N) management are the three most important agronomic measures for wheat (*Triticum aestivum* L.) production, but the combined effects on grain yield and wheat physiological characteristics are still poorly understood. We conducted a three-year split–split field experiment at the junction of the Loess Plateau and Huang-Huai-Hai Plain in China. The two irrigation practices (I0: non-irrigation and I1: one-off irrigation), three tillage methods (RT: rotary tillage, PT: plowing, and ST: subsoiling), and four N managements (N0, N120, N180, and N240) were assigned to the main plots, subplots, and sub-subplots, respectively. Irrigation practice, tillage method, N management, and most of their two-factor and three-factor interactions could significantly affect grain yield and the physiological characteristics of the leaves of winter wheat. One-off irrigation increased the grain yield by 46.9% by optimizing the activities of superoxide dismutase (SOD), peroxidase (POD) and catalase (CAT), the contents of proline (Pro) and soluble sugar (SS), and the net photosynthesis rate (Pn) in leaves during most growth stages of wheat. The improvement of grain yield and physiological characteristics under one-off irrigation was considerably affected by the tillage method and N management, and the effectiveness of one-off irrigation was improved under subsoiling and N180 or N240. One-off irrigation combining subsoiling and N180 had no significant difference relative to one-off irrigation combining subsoiling and N240, while it significantly increased grain yield by 47.1% over the three years, as well as increasing the activities of SOD, POD, and CAT, and Pn in wheat leaves by 23.2%, 41.2%, 26.1%, and 53.0%, respectively, and decreasing the contents of malondialdehyde (MDA), Pro, and SS by 29.2%, 65.4%, and 18.2% compared to non-irrigation rotary tillage combined with N240 across the two years and three stages. The wheat grain yield was significantly associated with the physiological characteristics in flag leaves, and the coefficient was greatest for POD activity, followed by SOD activity and Pn. Therefore, one-off irrigation combining subsoiling and N180 is an optimal strategy for the high-yield production of wheat in dryland regions where the one-off irrigation is assured.

## 1. Introduction

Wheat (*Triticum aestivum* L.) is one of the stable food crops and the world’s most widely grown crop, and its yield level plays a crucial role in ensuring global food security [1,2]. Despite this, around 75% of wheat production emanates from dryland regions in the world—zones mainly characterized as arid, semi-arid, and semi-humid drought-prone [3,4,5]. In these regions, due to the low and mismatching rainfall and the poor infrastructure, wheat is frequently subjected to drought dress, which inhibits many metabolic processes and makes it difficult for wheat to complete its life cycle, thus causing grain production potential to decrease by up to 60–70% [6,7,8,9,10,11,12]. Thus, the evaluation of the metabolic processes under different agronomic measures can help explain the causality behind changes in wheat productivity in dryland regions.

Under drought stress, reactive oxygen species (ROS) are produced in cells and trigger oxidative damage, such as lipid peroxidation of biofilms, protein denaturation, photosynthetic pigment loss, and impaired photosynthesis, ultimately resulting in a severe reduction in grain yield [12,13,14]. In fact, the frequent drought stress in dryland wheat production systems leads to ROS accumulation and a decline in plant photosynthetic capacity, resulting in an obvious reduction in wheat yield [7,10]. In order to survive, plants have developed a variety of physiological and biochemical adaptations to cope with drought stress [14]. Firstly, the ROS in cells can be removed to maintain the stability and function of the cell membrane structure by increasing the activity of the antioxidant enzyme system, such as superoxide dismutase (SOD), peroxidase (POD), catalase (CAT), etc. [12,14,15,16]. Increases of these enzymes in plants can boost grain yield by reducing ROS damage, and they are often used as physiological indicators for evaluating the response of wheat growth and development to stress [12,14]. Another important biochemical response to drought stress is osmoregulation, such as the accumulation of free proline (Pro) and soluble sugar (SS), which can buffer cellular redox potential to maintain biomolecule structural and physiological functions [14,16,17]. In addition, the role of plants in response to drought stress is multifaceted; many other physiological parameters have been used to assess the drought tolerance phenotype of wheat, including malondialdehyde (MDA) and Pn [12,14,15,16]. Recently, the physiological characteristics have been getting more attention for exploring how agronomic management contributes to wheat production and yield formation in dryland crop production systems [12,14,16,17].

Optimizing wheat physiological characteristics helps yield formation and increases wheat yield [12,14,18]. It is widely reported that suitable irrigation practices [19,20,21], tillage methods [18,22,23,24,25,26], N management [12,27], and their two-factor interactions [21,28,29,30] could affect wheat yield by regulating physiological characteristics. For instance, in the Loess Plateau of China, compared to other irrigation and simulated rainfall models, the 150 mm irrigation under 200 mm simulated rainfall model increased the activities of SOD, POD, and CAT but reduced the contents of MDA and Pro in flag leaves during different growth stages of winter wheat, thus achieving higher Pn and the highest grain yield [31]. Another study in Huang-Huai-Hai plain of China showed that irrigation based on the consideration of precipitation, soil water storage, and crop requirements with amounts of 51–57 mm could increase the activities of SOD and CAT but reduced the MDA content in flag leaves of wheat compared to non-irrigation, and thus significantly increased the grain yield by 16.7–32.2% [20]. In the North China Plain of China, irrigation with 30 or 45 mm at the jointing and anthesis stage also significantly increased the activities of CAT and POD in flag leaves, resulting in an increase in wheat yield [21]. As a conservation tillage, subsoiling can ameliorate soil structure properties and sustainability, increase soil water storage, change root distribution, promote deep soil water and N uptake by wheat, and finally achieve better plant physiological characteristics and grain yield [23,31,32,33,34]. Besides irrigation practice and tillage methods, exogenous N supply helps alleviate the abiotic stress in wheat plants by increasing the Pro content and antioxidant enzyme activities compared to N-untreated plants and significantly increases the grain yield of wheat [35]. Ru et al. also reported that scientific N management alleviates the negative impact of drought stress on wheat, resulting in a significant increase in grain yield by improving physiological characteristics and delaying plant senescence [12]. Xue also reported that scientific N management alleviates the negative impact of drought stress on wheat and resulting in a significant increase of grain yield by improving physiological characteristics and delaying plant senescence [36]. Considering the two-factor interaction, Wang et al. reported that irrigation practice and tillage methods had significant effects on wheat physiological characteristics and grain yield [23]. Du et al. also reported that optimum irrigation combined with optimum N treatment increased wheat yields due to increased activities of SOD and POD, SS content, and Pn in leaves at various stages, compared to a system that is deficient in irrigation and N treatment [28]. However, previous studies mainly focus on single-factor or two-factor effects of irrigation practices, tillage methods, and N management on the mechanism of regulating wheat grain yield by physiological characteristics, but the combined effect of these three factors remains unclear, particularly regarding the changes in antioxidative enzymes, osmotic regulatory substances, and net photosynthesis rate of wheat leaves in dryland regions.

Recently, the progression of the High-Standard Farmland Construction Program in China and around the world has been swift, which has cemented the provision of at least one-off irrigation (irrigate once during wheat growth stage) for wheat growth in numerous dryland regions—locales previously bereft of irrigation [5,14]. A field study in the study area showed that one-off irrigation significantly increased dryland wheat yield by 56%, and the increase could be enhanced by optimizing tillage methods and N management [37]. Therefore, it is an urgent necessity to refine irrigation and other agronomic tactics to improve physiological characteristics during wheat growth stages and to grasp this one-off irrigation opportunity to bolster grain yields in dryland regions where one-off irrigation is assured. Thus, the present study investigated the physiological mechanism of the combined one-off irrigation, subsoiling, and N management on wheat yields based on a three-year split–split plot field experiment (two irrigation practices, three tillage methods, and four N management) in a typical semi-humid drought-prone region in China. The objectives are to test the hypotheses that (1) one-off irrigation, subsoiling, and proper N management could effectively increase wheat grain yield by enhancing the capacity of ROS scavenging, cellular osmoregulation, and Pn in leaves; (2) the effects of irrigation practices, tillage methods, and N management on wheat leaf physiological characteristics and grain yield would have a synergistic positive effect; and (3) the combination of one-off irrigation, subsoiling, and N rate of 180 kg ha^−1^ could be a practice to improve wheat yield by optimizing leaf physiological characteristics in dryland regions.

## 2. Results

### 2.1. Grain Yield

**Figure 1:**
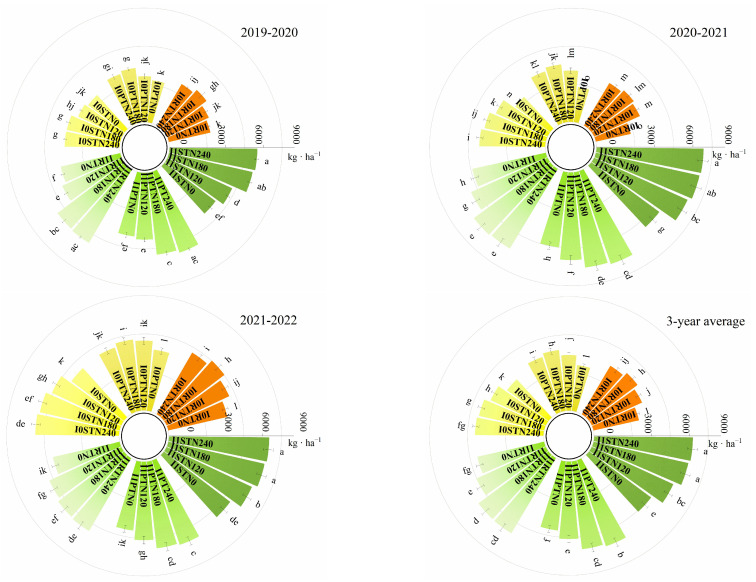
Effects of the interaction of irrigation practice, tillage method, and N management on grain yield in 2019–2022. Note: I0, no-irrigation; I1, one-off irrigation; RT, PT, and ST refer to rotary tillage, plowing, and subsoiling, respectively; N0, N120, N180, and N240 refer to N managements with the N rates of 0, 120, 180, and 240 kg N ha^−1^, respectively. Different lowercase letters above bars indicate significant differences by Duncan’s test at *p* < 0.05, and only the first and last letters are retained when there are more than two lowercase letters.

**Figure 2:**
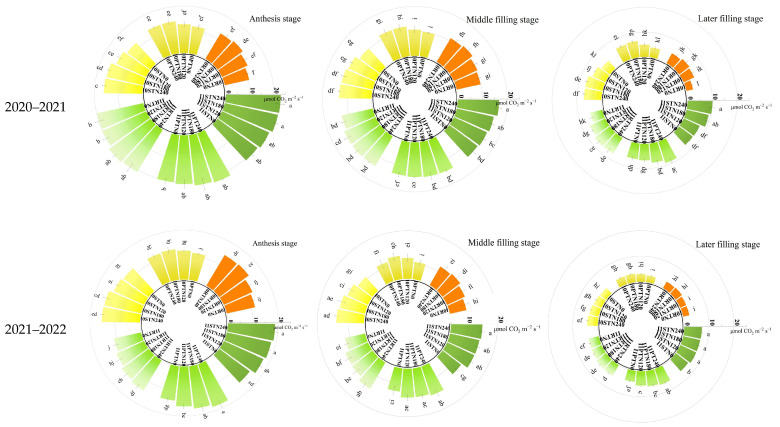
Effects of the interaction of irrigation practice, tillage method, and N management on Pn in wheat leaves in 2019–2022. I0, no-irrigation; I1, one-off irrigation; RT, PT, and ST refer to rotary tillage, plowing, and subsoiling, respectively; N0, N120, N180, and N240 refer to N managements with the N rates of 0, 120, 180, and 240 kg N ha^−1^, respectively. Different lowercase letters above bars indicate significant differences by Duncan’s test at *p* < 0.05, and only the first and last letters are retained when there are more than two lowercase letters.

Irrigation practices, tillage methods, and N management significantly affected grain yield in all the three years (Table 1, Figure 1). Averaged across the three years, one-off irrigation considerably increased grain yield by 46.9% compared to non-irrigation; subsoiling produced more grain yield with 11.5% higher yields than plowing and 13.5% higher than rotary tillage. There was no significant difference between N180 and N240 over the three years, while they produced a 10.7–28.4% (*p* < 0.05) higher grain yield than N0 and N120. Grain yield was affected by irrigation × N management in all three years, while by irrigation × tillage in one year, and by tillage × N management and irrigation × tillage × N management in two years (Table 2). These results showed that the improvement of grain yield in one-off irrigation was affected by tillage methods and N management, but the effects varied among years. As shown in (Figure 2), under non-irrigation, N180 produced a better grain yield than the other N managements under rotary tillage and plowing in all three years; however, N180 and N240 had the same high yield under subsoiling. Under one-off irrigation, N180 and N240 produced the same high grain yield, while all were significantly higher than N0 and N120 under all three tillage methods in all three years; likewise, subsoiling produced higher yields than plowing and rotary tillage under the same N managements. Thus, I1STN180 and I1STN240 had no significant yield difference, but they all significantly increased compared to other combinations except for I1RTN240 and I1PTN240 in 2019–2020, with an average increase of 7.0–89.3% across the three years. Therefore, I1STN180 was the best combination because it saved 25% N fertilizer without sacrificing grain yield compared to I1PTN240.

### 2.2. Activities of SOD, POD and CAT

As shown in (Table 2, Table 3 and Table 4), the activities of SOD, POD, and CAT in wheat leaves were considerably affected by irrigation practice in the two years and three growth stages, and they were also significantly affected by the tillage method and N management in most conditions. Moreover, the effectiveness of different treatments on SOD, POD, and CAT were similar over years and growth stages. On average, one-off irrigation significantly increased the activities of SOD, POD, and CAT by 19.4%, 31.3%, and 25.6%, respectively, compared to non-irrigation; subsoiling increased the activities of SOD, POD, and CAT by 5.7%, 9.4%, and 5.3%, respectively, compared to plowing, as well as by 7.7%, 8.5%, and 6.0%, respectively compared to rotary tillage. With the increase in N rate under the same irrigation practice and tillage method, the activities of SOD, POD, and CAT increased firstly and then stabilized with the inflection point at N180 in most conditions, and N180 and N240 were significantly higher than N0 and N120.

The two-factor and three-factor interactions of irrigation practice, tillage method, and N management considerably affected the activities of SOD, POD, and CAT during more than 50% of stages (Table 2, Table 3 and Table 4). This indicated that these three factors had certain interactions with the antioxidant enzyme activity of wheat leaves, and the effects were different over years and growth stages. Generally speaking, there was no significant difference in the activities of these antioxidant enzymes between I1STN180 and I1STN240 except for SOD activities during later filling stage in 2021–2022; however, I1STN180 significantly increased the activities of antioxidant enzymes in most cases compared to the other combinations, with the increase of 21.8%, 34.9%, and 25.8% in terms of SOD, POD, and CAT activities over the two years and three stages. Therefore, one-off irrigation, subsoiling, and N180 or N240 were the best combinations for increasing the activities of antioxidant enzymes in leaves of wheat in dryland.

### 2.3. MDA Content

The MDA contents in wheat leaves were significantly affected by irrigation practice, tillage method, and N management at all three growth stages in the two years (Table 5). Averaged across years and stages, one-off irrigation significantly decreased MDA content by 21.7% compared to non-irrigation; subsoiling decreased MDA content by 9.6% and 12.2% compared to plowing and rotary tillage, respectively. With the increase in N rate, the MDA content decreased firstly and then stabilized with the break point under N180 over the two years.

The MDA content was considerably affected by the interaction of irrigation practice × tillage method except for the middle filling stage in 2020–2021; it was also considerably affected by the interaction of irrigation practice × N management at the middle and later filling stages in the two years. However, the interaction of tillage method × N management was not significant during the middle and later filling stages in 2020–2021 and during the later filling stage in 2021–2022, and the three-factor interaction was only significant at one stage in the two years (Table 5). I1SSN180 significantly decreased in most cases compared to other combinations except for I1SSN240, with an average decrease of 22.3% across the two years and three stages.

### 2.4. Contents of Pro and SS

The contents of proline (Pro) and soluble sugar (SS) were considerably affected by irrigation practices, tillage methods, and N management during all three stages in the two years (Table 6 and Table 7). On average, one-off irrigation significantly decreased the contents of Pro and SS in leaves by 51.5% and 26.1%, respectively, compared to non-irrigation; however, subsoiling significantly increased by 18.3% and 11.9% compared to plowing, as well as by 24.0%, and 17.6% compared to rotary tillage. Under the same irrigation practice and tillage method, the contents of Pro and SS first increased and then stabilized with the increase in N rate. There was no significant difference between N180 and N240 over the two years, while they were significantly higher than N0.

The interaction effects of irrigation practice × tillage method and irrigation practice × N management on leaf Pro content were significant during two stages in 2020–2021 and all three stages in 2021–2022. The interaction on the SS content was not significant in all stages (except for the irrigation practice × N management interaction at later filling stage) in 2020–2021, but it was significant during all stages in 2021–2022. The interaction of tillage method × N management and the three-factor interaction on the contents of Pro and SS in leaves was significant at all the stages in 2021–2022 (Table 6 and Table 7). I0STN240 or I0STN180 had the highest contents of Pro and SS in wheat leaves, and I1SSN180 was increased by 52.9% and 27.4% compared to other combinations across the two years and three stages.

### 2.5. Pn

Irrigation practice, tillage method, and N management considerably affected the leaf Pn at all three growth stages in the two years, and the effects were the same during the different growth stages (Table 8). The mean of the three growth stages and two year results depicted that one-off irrigation significantly increased the Pn in leaves by 42.7% compared to non-irrigation; subsoiling respectively increased the Pn in wheat leaves by 10.9% and by 21.1% compared to plowing and rotary tillage. With the increase in N rate under the same irrigation, the Pn increased first and then stabilized compared to N0; the treatments with N fertilizer significantly increased Pn in leaves. However, in most conditions, there was no significant difference among N120, N180, and N240 during the three stages over the two years. The two-factor and three-factor interaction effects on Pn in leaves were not significant during most stages in the two years (Figure 2). These results indicated that there were synergistic positive effects of irrigation practices, tillage methods, and N management on leaf Pn in dryland wheat. Thus, I1SSN240 produced the highest Pn in leaves, but it had no significant difference in comparison to I1SSN180. I1SSN180 increased the Pn in leaves by 7.3–92.9% across the two years and three stages, compared to other combinations except for I1SSN240.

### 2.6. The Correlation Analysis of Physiological Characteristics and Yield

**Figure 3:**
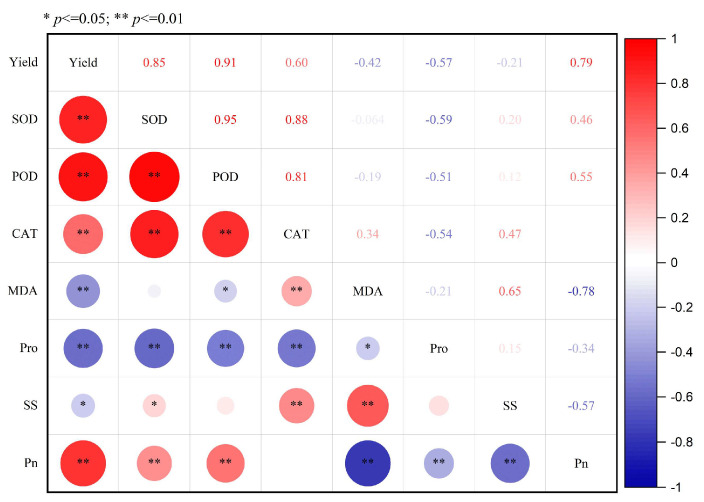
The correlation analysis between grain yield and physiological characteristics in leaves of wheat in dryland regions. Data are the mean of two years and three growth stages. * and ** are significant correlations at *p* < 0.05 and *p*< 0.01, respectively.

Regression analysis (Figure 3) showed a significant positive correlation between grain yield and the activities of SOD, POD, and CAT, and Pn (*p* < 0.01). There was a significant negative relation between yield and the contents of Pro and MDA (*p* < 0.01) and SS (*p* < 0.05). In conclusion, optimizing the antioxidant defense system, Pro content and Pn after flowering were beneficial to yield formation and promoted the increase in yield.

## 3. Discussion

### 3.1. One-Off Irrigation Combining Subsoiling and N180 Enhanced Wheat Yields in Dryland Regions

Increasing yields remains the main objective of current wheat production. In the dryland wheat production system in China, water can not meet the needs of wheat growth, which leads to severe wheat yield loss [36,38]. Many studies have explored the individual effects of irrigation practices, tillage methods, N management, and their interactions [20,39,40,41,42,43,44,45,46,47] on wheat yield and the mechanism of wheat yield formation. In the present study, irrigation practices, tillage methods, and N management can significantly affect wheat yield. One-off irrigation produced a better wheat yield related to non-irrigation, as well as subsoiling related to plowing and rotary tillage, and N180 or N240 related to N120 and N0. These results were in accordance with the previous studies [20,41,44]. Simultaneously, we found that any of the factors in terms of one-off irrigation, subsoiling, and N180 or N240 had synergistic positive effects on wheat yield when the third factor is fixed in dryland regions. Thus, the combination of one-off irrigation, subsoiling, and N180 or N240 achieved the highest yields. This was likely due to the best soil moisture and soil properties introduced by the one-off irrigation and subsoiling, but also the good N supply from the N180 or N240 N management; both of these benefits help provide suitable conditions for plant growth and development, tiller differentiation, and carbon and N metabolism, physiological progress, ultimately resulting in a high yield of wheat in dryland regions [20,39,40,41,42,43,44,45,46]. These results indicated that the wheat yield in dryland regions could be enhanced by the coordinated application of one-off irrigation, subsoiling, and N180 in the study area while saving 25% N input related to I1STN240. Other studies have also reported that wheat yield was enhanced by the interactions of irrigation practices with tillage methods [23], irrigation practices with N management [21,28], and tillage methods with N management.

### 3.2. One-Off Irrigation Combining Subsoiling and N180 Could Enhance Physiological Characteristics in Wheat Leaves

Antioxidant traits (including SOD, POD, and CAT enzymes) for crop plants play an important role in scavenging oxidative damage under stress, which is helpful to yield formation [16]. Malondialdehyde (MDA)—a secondary product of membrane lipid peroxidation—is usually used to indicate the degree of oxidative damage suffered by the membrane system under stress [23]. Our results showed that one-off irrigation decreased the SOD, POD, and CAT activities but increased MDA content in wheat leaves compared to non-irrigation, indicating that wheat plants under non-irrigation treatment were suffering from oxidative damage, and one-off irrigation can alleviate this oxidative damage. Likewise, subsoiling decreased the SOD, POD, and CAT activities but increased MDA content in wheat leaves in comparison to plowing and rotary tillage and N180 or N240 in comparison to N0. These results suggest that one-off irrigation, subsoiling, and N180 help optimize the antioxidant system in wheat leaves after plowing, thus assuring wheat growth by increasing antioxidant enzyme activities and decreasing MDA content. The improvement of antioxidant traits in wheat leaves was also reported by irrigation [16], subsoiling [23], and proper N management [48,49]. Osmoregulation is an important mechanism underlying drought resistance in plants [16,50]. Pro and SS are the important indicators that reflect the degree of osmoregulation [16]. Our results showed that the contents of Pro and SS in wheat leaves decreased under non-irrigation in comparison to one-off irrigation, indicating that wheat plants suffered less stress during the grain-filling stage under one-off irrigation. A report by [51] showed that plant cells can stabilize their intracellular environment and pressure potential by accumulating low-molecular-weight organic compounds, such as Pro and SS, thereby alleviating the harm induced by drought stress. In the present study, subsoiling increased the contents of Pro and SS in wheat leaves compared to rotary tillage or plowing. Likewise, an increased N application rate also increased the contents of Pro and SS in wheat leaves, regardless of irrigation practices and tillage methods (Table 5 and Table 6). These results indicated that the subsoiling and increased N rate could help to enhance wheat cellular osmoregulation by increasing the contents of Pro and SS in wheat leaves and alleviating the harm of drought stress. Previous studies conducted in dryland regions also showed that subsoiling and proper N management increased the Pro contents of wheat [39].

Pn in plant leaves can represent the photosynthetic characteristics of winter wheat [18]. Drought stress reduced photosynthesis and accelerated leaf senescence, shortening the grain-filling duration of crops thereby reducing grain yield [16,52]. Previous studies have demonstrated that irrigation practices, tillage methods, and N management affect the soil temperature, water regime, and other soil physical-chemical properties, which are very important for providing favorable nutritional conditions for the photosynthesis process [16,18,52,53]. The results in our trial also showed that one-off irrigation, subsoiling, and N180 or N240 were prone to increase Pn in wheat leaves, and these three tactics have synergistic effects on Pn in wheat. Thus, the combination of one-off irrigation, subsoiling, and N180 or N240 was prone to increase leaf Pn during the whole grain filling stage in dryland regions. The present study also revealed that Pn was positively correlated with wheat grain yield, with R only lower than SOD and POD activities; this indicated that the Pn in flag leaves was a major factor in decreasing grain yield (Figure 3). However, in this study, we only measured the Pn in the flag leaves. In future studies, the effects of different irrigation practices, tillage methods, and N management on the canopy photosynthetic characteristics, such as light capturing ability and light energy utilization efficiency, should also be studied.

The present study further confirmed the common perception that one-off irrigation after regreening considerably improves wheat yield by optimizing physiological characteristics in dryland regions compared to non-irrigation, and the improvements were greatly affected by the tillage methods and N management. We concluded that one-off irrigation combined with subsoiling and N180 performed much better than other combinations in terms of grain yield and physiological characteristics in leaves after flowering. Thus, this study has provided new academic insights into how to manipulate tillage methods and N management to increase the benefits of irrigation in dryland regions where one-off irrigation is assured. However, different winter wheat varieties may show different responses to different irrigation practices, tillage methods, and N management. Only one winter wheat variety was tested in this study; many different varieties have been developed in the dryland wheat production regions. For this reason, this topic requires further exploration.

## 4. Materials and Methods

### 4.1. Experimental Site Description

**Figure 4:**
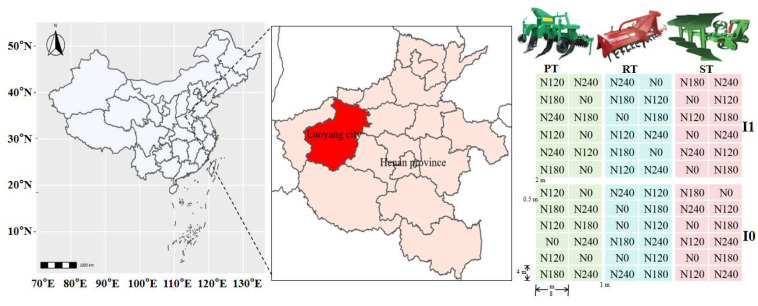
Information of experimental site and treatment layout in field. I0, no-irrigation; I1, one-off irrigation; RT, PT, and ST refer to rotary tillage, plowing, and subsoiling, respectively; N0, N120, N180, and N240 refer to N managements with the N rates of 0, 120, 180, and 240 kg N ha^−1^, respectively.

**Figure 5:**
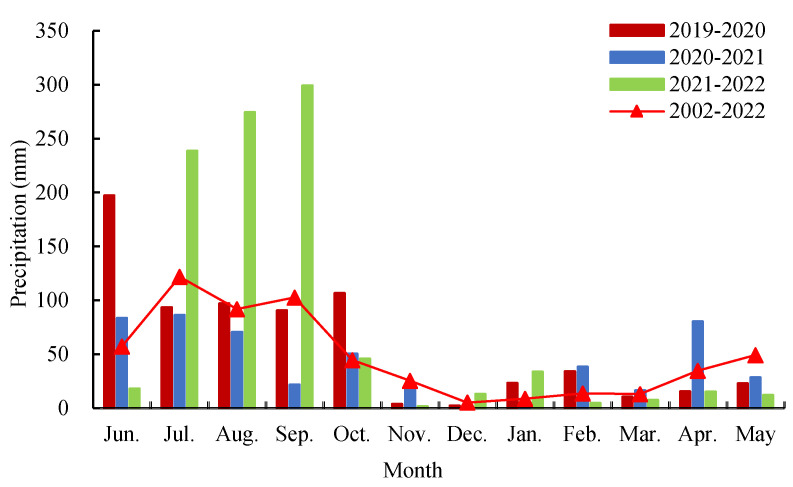
Monthly precipitation during the experiment years of 2019–2020, 2020–2021, 2021–2022, and the average from 2002 to 2022.

A field experiment was performed from October 2019 to June 2022 at the Dryland Experimental Station of Henan University of Science and Technology (34°44′ N, 112°40′ E), which is located in the Dugou village, Yaling town, Yichuan county, Luoyang city, Henan Province, China (Figure 4). The experimental site is characterized by a semi-arid and temperate continental monsoon climate. The annual average temperature was 14.5 °C, with an average precipitation of 633.4 mm, with >60% of rainfall concentrated from June to September (Figure 5). The frost-free period was about 235 days, and the number of sunshine hours was 2311 h. The soil was classified as heavy loam according to the FAO (1993) [54]. At the initiation of the experiment in 2019, the basic properties in the 0–20 cm soil layer were as follows: field capacity of 27.4%, bulk density of 1.35 g cm^−3^, organic matter content of 14.7 g kg^−1^, total N content of 1.11 g kg^−1^, available phosphorus content of 9.0 mg kg^−1^, available potassium content of 139.6 mg kg^−1^, and pH of 7.57.

### 4.2. Experimental Design

The experiment was conducted using a split–split plot design with irrigation practice as the main plot treatment, tillage methods as the subplot treatment, and N management as the split–split subplot treatment (Figure 4). Two irrigation practices were laid out in the experiment: non-irrigation (I0) and one-off irrigation (I1, irrigated to 85% of field capacity when the soil water content in the 0–40-cm soil layer was lower than 60% of field capacity at the first time after wheat regreening). The three tillage methods were rotary tillage (RT), plowing (PT), and subsoiling (ST). The four N managements were N0, N120, N180, and N240, where N rates were 0, 120, 180, and 240 kg·hm^−2^, respectively. The N management varied depending on irrigation practice; all N was applied at sowing under non-irrigation treatment, 50% as basal, and the other 50% were applied along with irrigation under one-off irrigation treatment. There were three replications for each treatment, and the plot area was 32 m^2^ (4 m × 8 m).

The irrigation amount was calculated using the following equation [55]:

IA = 10 × ρb × H × (β_i_ − β_j_),

where IA is the irrigation amount after wheat regreening; ρb is the average soil bulk density (g cm^−3^) in the planned wetting layer (0–40 cm); H is the depth (cm) in the planned wetting layer (0–40 cm); β_i_ is the average value of the target soil water content in the 0–40 cm soil layer (%); and β_j_ is the average soil water content in the 0–40 cm soil layer before irrigation. The irrigation amount was controlled by a water meter.

Tillage methods were conducted 3–5 days before wheat sowing. For rotary tillage treatment, a rotavator was used with a depth of 10–15 cm. For plowing treatment, the soil was plowed to a depth of 30–35 cm using a moldboard plow, and then a rotavator was used to tidy up the seedbed. Rotary tillage and plowing were implemented each year. For subsoiling treatment, the soil was loosened (35–40 cm in depth, 35 cm of distance) using a subsoiling chisel; then, a rotavator was used to tidy up the seedbed. Subsoiling was implemented in 2019–2020 and 2021–2022, and rotary tillage was carried out in 2020–2021.

For non-irrigation, all N fertilizer, phosphorus (P) fertilizer (90 kg P_2_O_5_ ha^−1^), and potassium (K) fertilizer (60 kg K_2_O ha^−1^) were manually broadcast in the corresponding plot and thoroughly incorporated into the soil by rotary tillage. For one-off irrigation, 50% N fertilizer, 90 kg P_2_O_5_ ha^−1^, and 60 kg K_2_O ha^−1^ were manually broadcast in the corresponding plot and thoroughly incorporated into the soil by rotary tillage; the other 50% N fertilizer was manually applied with irrigation. The N, P, and K fertilizers were urea (N, 46%), triple superphosphate (P_2_O_5_, 12%), and potassium sulfate (K_2_O, 50%), respectively. Wheat (*Triticum aestivum* L.) cultivar ‘Luohan 22′ was used in the study. In each year, wheat was sown in middle or late October at a seeding rate of 187.5–225.0 kg ha^−1^ and harvested in late May or early June. Weeds, pests, and diseases were controlled with herbicides and pesticides according to local practices.

### 4.3. Measurements and Methods

#### 4.3.1. Grain Yield

At maturity (Zadoks 92) in 2019–2022, four random areas (1 m × 1 m) in each plot were harvested by hand to measure grain yield (kg ha^−1^). After air drying, the samples in each plot were threshed, and 50 ± 5 g grains were oven-dried at 70 °C to a constant weight. Grain moisture was measured, and the yield (kg ha^−1^) was calculated based on a grain moisture content of 12.5%.

#### 4.3.2. Antioxidant Defense System and Osmotic Substances

In 2020–2021 and 2021–2022, forty flag leaves were randomly sampled in the central position of each plot at anthesis (Zadoks 65), middle filling (Zadoks 75), and later filling (Zadoks 85). The samples were treated with liquid N for 20 min and kept at −80 °C until their subsequent analysis for the activities of SOD, POD, and CAT and the contents of MDA, Pro, and SS. SOD enzyme activity (U g^−1^ FW) was assayed according to [56]. POD activity (U g^−1^ FW min^−1^) was assayed by the guaiacol method proposed by [57]. CAT activity (U g^−1^ FW min^−1^) was determined using the permanganate titration method [58]. MDA content (μmol g^−1^ FW) was determined by the thiobarbituric acid method [59]. Pro content (mg g^−1^ FW) was assayed by the ninhydrin colorimetric method [60]. SS content (mg g^−1^ FW) was determined using anthrone colorimetry [61].

#### 4.3.3. Net Photosynthesis Rate (Pn)

At the anthesis (Zadoks 65), middle filling (Zadoks 73), and later filling (Zadoks 83) stages, the Pn in flag leaves was measured using a Portable Photosynthesis System (LI-Cor 6400XT, Lincoln, NE, USA) equipped with a red and blue LED leaf chamber. Measurements were taken at a CO_2_ concentration of 380 mmol mol^−1^ and a PPFD of 1100 μmol m^−2^ s^−1^ on sunny days in the morning (9:00–11:00 a.m.) to avoid potential stomatal closure of leaves, and the leaf chamber was maintained at the same temperature in the same growth stage. Each time, three flag leaves from three different wheat plants in the center of each plot were measured at the same leaf position, avoiding major veins.

### 4.4. Statistical Analysis

Data processing was performed using Microsoft Excel 2010 and SPSS 22 (IBM Corporation, New York, NY, USA). Differences in indicators among treatments were verified using Duncan’s test at *p* < 0.05. The graphical presentation and regression analysis were generated using Origin 2024 software (Origin Lab Corporation, Northampton, MA, USA).

## 5. Conclusions

Compared to non-irrigation, one-off irrigation considerably increased grain yield, which was attributed to improved activities of SOD, POD, and CAT, and Pn, and decreased contents of MDA, Pro, and SS in wheat leaves. The improvement in grain yield and leaf physiological characteristics under one-off irrigation was significantly affected by tillage methods and their interaction with N management in most cases. Thus, one-off irrigation combined with subsoiling and N180 or N240 increased the activities of antioxidant enzymes and Pn in wheat leaves and thus provided a favorable wheat yield compared to other combinations. These results indicated that one-off irrigation combined with subsoiling and N rate of 180 with 50% as basel and 50% topdressing with the irrigation was an alternative strategy to achieve high yields by improving physiological characteristics in wheat leaves in dryland regions. This study provides a global sample to increase wheat yield through the manipulation of tillage and N fertilizer in dryland regions where one-off irrigation was assured.

## Figures and Tables

**Table 1:** Grain yield (kg ha^−1^) of wheat affected by irrigation practice, tillage method, and N management in 2019–2022.

Treatment	2019–2020	2020–2021	2021–2022	Average
I0	3885.0 b	4018.0 b	5640.0 b	4514.0 b
I1	5980.0 a	7216.0 a	6700.0 a	6632.0 a
RT	4819.0 b	5228.0 c	5865.0 b	5304.0 b
PT	4869.0 b	5503.0 b	5822.0 b	5398.0 b
ST	5110.0 a	6121.0 a	6823.0 a	6018.0 a
N0	4222.0 c	4558.0 c	5391.0 c	4724.0 c
N120	4556.0 b	5647.0 b	6176.0 b	5460.0 b
N180	5481.0 a	6138.0 a	6578.0 a	6044.0 a
N240	5471.0 a	6125.0 a	6536.0 a	6066.0 a
I	1279.1 **	5988.2 **	4019.8 **	17737.7 **
T	16.8 **	147.4 **	310.5 **	252.3 **
N	377.0 **	431.8 **	198.6 **	870.3 **
I × T	0.25 ns	2.9 ns	23.7 **	11.0 **
I × N	71.8 **	3.8 *	6.8 **	32.2 **
T × N	1.3 ns	3.4 **	2.9 *	8.3 **
I × T × N	2.2 ns	5.1 **	4.9 **	4.3 **

Note: I0, no-irrigation; I1, one-off irrigation; RT, PT, and ST refer to rotary tillage, plowing, and subsoiling, respectively; N0, N120, N180, and N240 refer to N managements with the N rates of 0, 120, 180, and 240 kg N ha^−1^, respectively. Different lowercase letters within columns indicate significant differences by Duncan’s test at *p* < 0.05. *, significant at *p* < 0.05; **, significant at *p* < 0.01; ns, not significant at *p* < 0.05.

**Table 2:** Effects of irrigation practices, tillage methods, and N management on SOD activity (U g^−1^ FW) in wheat leaves during different growth stages in 2020–2022.

Treatment			2020–2021			2021–2022		
			Anthesis Stage	Middle Filling Stage	Later Filling Stage	Anthesis Stage	Middle Filling Stage	Later Filling Stage
I0	RT	N0	408.9 h	373.2 l	311.2 j	299.6 jk	276.1 l	153.6 k
		N120	442.8 g	418.4 j	379.5 g	325.4 i	306.5 jk	215.4 hi
		N180	473.0 e	454.1 gh	436.2 df	330.9 hi	323.1 gh	251.5 g
		N240	466.9 ef	439.3 hi	430.8 ef	336.1 hi	325.4 gh	254.8 g
	PT	N0	406.9 h	396.2 k	336.6 i	288.0 k	272.8 l	143.4 k
		N120	445.8 g	432.5 ij	385.9 g	323.5 ij	301.3 k	197.6 i
		N180	534.0 d	500.4 df	447.6 ce	326.6 i	317.7 hj	265.0 g
		N240	530.4 d	490.3 ef	446.6 ce	331.9 hi	320.0 gi	266.7 g
	ST	N0	444.4 g	419.5 j	336.1 i	324.2 i	281.4 l	177.2 j
		N120	449.6 fg	441.2 hi	377.4 g	353.5 fh	320.2 gi	227.6 h
		N180	544.5 bd	518.1 cd	456.4 ac	364.4 eg	344.3 ef	294.7 f
		N240	541.0 cd	512.0 cd	450.2 bd	365.6 df	351.3 de	300.6 ef
I1	RT	N0	531.1 d	449.7 gi	351.2 hi	369.8 df	305.1 jk	213.4 hi
		N120	559.2 ac	492.9 ef	425.4 f	388.5 ce	343.6 ef	311.5 ef
		N180	561.8 ab	508.6 ce	470.8 a	397.3 bc	352.3 de	366.4 c
		N240	567.3 a	491.1 ef	463.8 ac	397.1 bc	359.6 cd	360.0 cd
	PT	N0	531.3 d	468.2 g	369.1 gh	341.1 gi	303.5 k	253.0 g
		N120	570.9 a	502.8 cf	430.4 ef	389.3 cd	331.9 fg	308.7 ef
		N180	574.5 a	538.0 ab	468.5 a	402.3 bc	358.7 cd	347.9 d
		N240	576.7 a	540.0 a	463.0 ac	404.9 ac	367.4 bc	342.4 d
	ST	N0	536.7 d	487.4 f	378.7 g	381.8 ce	307.6 ik	309.0 ef
		N120	569.2 a	520.8 bc	437.7 df	398.5 bc	348.8 de	318.0 e
		N180	576.9 a	541.4 a	466.7 ab	421.2 ab	374.9 ab	394.3 b
		N240	577.1 a	544.3 a	464.0 ac	426.9 a	381.6 a	418.7 a
I			1766.6 **	948.4 **	2122.4 **	166.2 **	8168.7 **	622.1 **
T			26.1 **	126.7 **	9.6 **	58.3 **	49.8 **	993.3 **
N			190.0 **	184.1 **	386.4 **	2.5 ns	2.6 ns	39.0 **
I × T			10.5 **	2.8 ns	1.4 ns	81.3 **	178.8 **	1469.3 **
I × N			53.3 **	12.5 **	7.1 **	0.8 ns	0.8 ns	0.0 ns
T × N			7.5 **	4.4 **	1.3 ns	2.4 ns	2.3 ns	22.7 **
I × T × N			5.4 **	0.9 ns	1.1 ns	1.1 ns	0.5 ns	25.7 **

I0, no-irrigation; I1, one-off irrigation; RT, PT, and ST refer to rotary tillage, plowing, and subsoiling, respectively; N0, N120, N180, and N240 refer to N managements with the N rates of 0, 120, 180, and 240 kg N ha^−1^, respectively. Different lowercase letters within columns indicate significant differences by Duncan’s test at *p* < 0.05, and only the first and last letters are retained when there are more than two lowercase letters, **, significant at *p* < 0.01; ns, not significant at *p* < 0.05.

**Table 3:** Effects of irrigation practices, tillage methods, and N managements on POD activity (U g^−1^ FW min^−1^) in wheat leaves during different growth stages in 2020–2022.

Treatment			2020–2021			2021–2022		
			Anthesis Stage	Middle Filling Stage	Later Filling Stage	Anthesis Stage	Middle Filling Stage	Later Filling Stage
I0	RT	N0	16.7 j	16.8 m	9.1 j	10.5 k	11.7 k	8.4 jk
		N120	18.5 gi	21.2 k	10.6 hi	13.5 ij	15.0 hi	9.1 ik
		N180	19.9 eg	23.2 ij	12.9 ef	15.5 eh	15.4 fh	11.5 fg
		N240	20.9 e	22.2 jk	12.8 ef	14.5 fi	15.2 gh	9.4 hj
	PT	N0	17.3 ij	17.8 lm	9.5 j	7.6 l	9.3 l	7.6 k
		N120	19.2 fh	21.2 jk	10.0 ij	13.3 ij	14.7 hj	8.6 jk
		N180	20.0 ef	25.9 gh	13.6 de	15.6 dg	15.5 fh	10.6 gi
		N240	19.2 fh	26.4 fh	13.4 de	14.4 gi	15.0 hi	8.9 ik
	ST	N0	18.0 hj	18.9 l	9.5 j	12.4 j	13.5 j	8.6 jk
		N120	19.4 fh	21.5 jk	11.0 gi	14.3 hi	16.5 ef	9.4 hj
		N180	20.1 ef	26.8 eh	13.5 de	15.5 eh	17.5 ce	12.7 ef
		N240	19.9 eg	28.0 df	13.2 e	15.6 dg	18.0 bd	12.9 ef
I1	RT	N0	22.8 d	22.5 jk	10.7 hi	15.7 df	16.4 eg	11.5 fg
		N120	26.7 bc	25.7 gh	12.1 fg	16.5 ce	17.4 ce	14.0 de
		N180	28.9 a	30.8 bc	14.7 c	16.8 bd	18.2 bc	14.7 cd
		N240	28.3 a	31.4 ab	15.0 ac	17.5 bc	18.4 bc	15.4 cd
	PT	N0	23.1 d	25.1 hi	11.1 gh	15.0 fh	13.8 ij	10.9 gh
		N120	26.3 c	28.6 de	13.1 ef	15.7 df	16.5 ef	11.5 fg
		N180	28.5 a	30.9 bc	14.9 bc	17.2 bc	18.4 bc	16.3 bc
		N240	28.1 ab	31.1 ac	14.4 cd	18.0 b	18.8 ab	17.5 ab
	ST	N0	23.9 d	27.4 dg	11.0 gi	16.7 ce	16.9 de	11.9 fg
		N120	27.7 ac	29.3 cd	12.9 ef	17.7 bc	17.9 bd	14.9 cd
		N180	29.1 a	32.9 a	15.8 ab	19.5 a	19.0 ab	17.8 ab
		N240	28.7 a	32.2 ab	16.1 a	20.7 a	19.8 a	18.2 a
I			7292.1 **	678.5 **	203.0 **	1986.1 **	667.2 **	360.4 **
T			2.2 ns	48.2 **	4.9 *	29.6 **	36.3 **	38.3 **
N			106.4 **	165.3 **	264.9 **	110.4 **	127.0 **	93.8 **
I × T			0.2 ns	0.7 ns	0.6 ns	2.2 ns	3.9 ns	1.6 ns
I × N			11.6 **	0.9 ns	0.8 ns	17.9 **	9.8 **	11.6 **
T × N			1.4 ns	0.7 ns	0.7 ns	3.7 ns	6.1 **	4.2 **
I × T × N			0.8 ns	5.5 **	2.9 *	4.8 **	0.3 ns	3.8 **

I0, no-irrigation; I1, one-off irrigation; RT, PT, and ST refer to rotary tillage, plowing, and subsoiling, respectively; N0, N120, N180, and N240 refer to N managements with the N rates of 0, 120, 180, and 240 kg N ha^−1^, respectively. Different lowercase letters within columns indicate significant differences by Duncan’s test at *p* < 0.05, and only the first and last letters are retained when there are more than two lowercase letters *, significant at *p* < 0.05; **, significant at *p* < 0.01; ns, not significant at *p* < 0.05.

**Table 4:** Effects of irrigation practices, tillage methods, and N managements on CAT activity (U g^−1^ FW min^−1^) in wheat leaves during different growth stages in 2020–2022.

Treatment			2020–2021			2021–2022		
			Anthesis Stage	Middle Filling Stage	Later Filling Stage	Anthesis Stage	Middle Filling Stage	Later Filling Stage
I0	RT	N0	329.0 hi	269.3 k	224.6 k	575.0 n	583.9 m	294.1 o
		N120	340.9 eg	337.9 gh	252.1 j	658.2 lm	665.6 jk	430.5 l
		N180	369.0 cd	365.7 e	286.5 hi	737.8 ij	815.7 de	561.6 j
		N240	364.7 cd	358.6 ef	284.2 hi	726.8 j	767.5 g	553.6 j
	PT	N0	317.0 j	308.7 j	230.8 k	552.7 o	566.6 m	268.7 p
		N120	337.9 fh	328.0 hi	292.5 gi	644.1 m	650.7 kl	398.8 m
		N180	366.2 cd	363.9 e	326.7 e	730.0 ij	735.2 hi	564.2 j
		N240	361.4 d	358.9 ef	322.7 ef	700.0 k	724.5 i	555.7 j
	ST	N0	325.0 ij	310.8 ij	280.5 i	582.8 n	637.0 l	362.4 n
		N120	349. 6 e	343.8 fh	326.3 e	669.5 l	688.9 j	517.8 k
		N180	373.8 c	371.3 e	350.3 cd	732.0 ij	783.1 fg	568.2 j
		N240	368.5 cd	362.9 e	352.4 c	745.7 i	803.4 ef	586.6 i
I1	RT	N0	336.5 fh	355.7 eg	298.3 gh	674.3 l	778.4 g	609.9 h
		N120	370.5 cd	445.2 d	346.0 cd	817.7 fg	817.8 de	618.5 fh
		N180	426.0 b	470.2 ac	372.4 ab	827.6 ef	862.9 bc	633.7 df
		N240	433.6 ab	468.7 ac	368.2 b	852.4 cd	873.4 b	643.4 de
	PT	N0	343.0 ef	359.2 ef	303.3 g	669.3 l	759.5 gh	603.1 h
		N120	368.2 cd	453.1 cd	336.1 de	803.4 gh	808.3 e	613.9 gh
		N180	433.7 ab	478.2 ab	380.2 ab	863.2 c	876.7 b	667.5 c
		N240	437.6 a	473.5 ab	384.5 a	863.4 c	884.5 b	678.2 bc
	ST	N0	331.7 gi	369.7 e	308.1 fg	798.6 h	806.3 ef	627.9 eg
		N120	365.2 cd	460.7 bd	337.7 ce	837.3 de	838.3 cd	649.4 d
		N180	434.7 ab	483.8 a	387.0 a	918.9 b	941.5 a	688.5 ab
		N240	437.2 a	479.1 a	384.9 a	938.1 a	958.0 a	697.4 a
I			6397.7 **	2783.8 **	926.7 **	112325.4 **	6889.5 **	11391.0 **
T			0.6 ns	5.9 *	103.0 **	235.0 **	139.7 **	93.2 **
N			863.5 **	426.0 **	317.6 **	106.9 **	18.2 **	13.3 **
I × T			3.4 ns	0.0 ns	62.2 **	5.4 **	15.8 **	627.7 **
I × N			139.0 **	34.0 **	1.3 ns	794.9 **	390.9 **	1532.0 **
T × N			1.4 ns	2.6 *	2.1 ns	9.5 **	3.0 **	22.2 **
I × T × N			1.5 ns	3.0 *	2.9 **	7.9 **	6.2 **	19.1 **

I0, no-irrigation; I1, one-off irrigation; RT, PT, and ST refer to rotary tillage, plowing, and subsoiling, respectively; N0, N120, N180, and N240 refer to N managements with the N rates of 0, 120, 180, and 240 kg N ha^−1^, respectively. Different lowercase letters within columns indicate significant differences by Duncan’s test at *p* < 0.05, and only the first and last letters are retained when there are more than two lowercase letters. *, significant at *p* < 0.05; **, significant at *p* < 0.01; ns, not significant at *p* < 0.05.

**Table 5:** Effects of irrigation practices, tillage methods, and N managements on MDA content (μmol g^−1^ FW) in wheat leaves during different growth stages in 2020–2022.

Treatment			2020–2021			2021–2022		
			Anthesis Stage	Middle Filling Stage	Later Filling Stage	Anthesis Stage	Middle Filling Stage	Later Filling Stage
I0	RT	N0	20.3 ab	29.4 a	39.4 a	25.5 b	41.1 b	49.7 a
		N120	21.4 a	25.4 ab	39.9 a	23.0 bd	28.0 c	38.9 c
		N180	17.7 c	24.2 bc	35.3 bc	19.9 eh	22.7 e	34.9 ef
		N240	17.1 cd	23.6 bd	36.5 ab	18.9 gi	22.2 ef	35.6 de
	PT	N0	19.4 b	27.8 dh	33.4 bf	30.6 a	46.5 a	51.0 a
		N120	15.0 gi	26.7 ei	29.5 fj	24.7 bc	28.3 c	44.0 b
		N180	15.4 eh	25.6 fj	30.3 di	22.1 ce	22.6 e	37.3 ce
		N240	16.1 dg	22.9 fj	30.1 di	21.1 dg	22.2 ef	36.8 ce
	ST	N0	17.1 cd	24.4 ce	32.4 cf	22.7 cd	28.5 c	45.6 b
		N120	15.8 dg	24.6 cf	27.5 hk	19.5 ei	25.3 d	37.9 cd
		N180	15.8 dg	22.1 dg	27.3 hk	19.2 fi	20.2 fi	32.6 f
		N240	15.5 dh	21.3 dh	28.2 gk	18.1 hj	20.0 gi	32.9 f
I1	RT	N0	16.7 cf	23.2 gk	33.9 bd	21.2 dg	22.2 ef	29.4 gh
		N120	16.9 ce	22.9 hl	31.6 cg	16.2 jl	21.5 eh	26.3 ik
		N180	14.9 gi	20.8 im	30.3 di	15.9 jl	18.2 ik	24.8 jl
		N240	14.1 hj	20.4 im	30.6 dh	14.6 kn	16.6 kl	23.9 km
	PT	N0	16.0 dg	21.9 km	33.8 be	21.5 df	21.9 eg	30.0 g
		N120	15.2 fi	21.0 lm	29.9 ej	17.1 ik	21.6 eh	27.5 hi
		N180	13.7 ij	20.0 lm	26.6 il	15.1 km	17.4 jl	24.0 km
		N240	13.0 j	19.9 m	24.8 kl	14.5 kn	16.7 kl	23.3 ln
	ST	N0	15.8 dg	20.3 jm	33.0 bf	18.0 hj	19.5 hj	28.6 gi
		N120	15.2 fi	19.7 km	26.1 jl	13.7 ln	18.4 ik	26.8 ij
		N180	13.2 j	19.7 km	25.0 kl	12.8 mn	16.8 kl	21.5 mn
		N240	13.8 ij	19.0 km	22.9 l	12.5 n	15.8 l	20.8 n
I			272.9 **	312.1 **	26.3 *	119.6 **	837.4 **	2635.1 **
T			31.4 **	17.9 **	105.6 **	201.4 **	82.5 **	23.5 **
N			34.6 **	22.2 **	30.7 **	165.2 **	374.2 **	238.2 **
I × T			5.7 *	2.2 ns	11.9 **	27.4 **	23.0 **	5.7 *
I × N			1.3 ns	3.0 *	3.3 *	1.4 ns	136.0 **	33.7 *
T × N			4.9 **	1.1 ns	2.0 ns	4.0 **	21.7 **	1.8 ns
I × T × N			3.1 *	1.2 ns	1.7 ns	2.1 ns	17.0 **	1.3 ns

I0, no-irrigation; I1, one-off irrigation; RT, PT, and ST refer to rotary tillage, plowing, and subsoiling, respectively; N0, N120, N180, and N240 refer to N managements with the N rates of 0, 120, 180, and 240 kg N ha^−1^, respectively. Different lowercase letters within columns indicate significant differences by Duncan’s test at *p* < 0.05, and only the first and last letters are retained when there are more than two lowercase letters *, significant at *p* < 0.05; **, significant at *p* < 0.01; ns, not significant at *p* < 0.05.

**Table 6:** Effects of irrigation practices, tillage methods, and N management on Pro content (mg g^−1^ FW) in wheat leaves during different growth stages in 2020–2022.

Treatment			2020–2021			2021–2022		
			Anthesis Stage	Middle Filling Stage	Later Filling Stage	Anthesis Stage	Middle Filling Stage	Later Filling Stage
I0	RT	N0	2401.4 f	296.7 jk	201.0 hj	197.9 hi	220.1 l	132.7 h
		N120	2474.9 f	324.6 ij	282.7 de	209.6 g	381.6 e	216.6 f
		N180	2782.7 e	423.8 d	393.4 ab	285.2 d	481.3 b	471.0 b
		N240	2555.8 f	405.0 de	382.1 ab	242.4 e	456.0 c	454.5 c
	PT	N0	3332.2 d	390.6 eg	232.3 ej	187.4 i	222.7 l	105.1 i
		N120	3512.9 bd	458.6 c	317.5 cd	208.7 gh	333.1 fg	144.2 g
		N180	3629.9 ac	530.2 a	412.3 a	223.5 f	439.7 d	417.2 d
		N240	3690.8 ab	536.1 a	389.4 ab	209.7 g	429.6 d	388.3 e
	ST	N0	3472.6 cd	413.3 de	260.9 ef	204.4 gh	269.6 k	143.6 gh
		N120	3527.1 bc	500.8 b	340.0 bc	328.2 c	386.6 e	223.2 f
		N180	3605.7 ac	548.2 a	408.9 a	364.9 b	518.8 a	488.5 a
		N240	3769.4 a	545.5 a	426.1 a	378.0 a	533.3 a	497.3 a
I1	RT	N0	108.7 g	273.9 k	136.7 k	49.1 o	133.1 o	26.5 p
		N120	194.7 g	320.9 j	187.6 ik	121.7 m	169.4 mn	49.4 mn
		N180	202.7 g	366.3 gh	234.7 ej	158.6 k	296.0 j	80.0 l
		N240	212.3 g	372.8 fh	238.4 ei	160.6 jk	316.6 hi	86.6 kl
	PT	N0	131.4 g	284.5 k	182.9 jk	47.3 o	127.5 o	22.6 p
		N120	193.0 g	368.7 gh	205.8 gj	113.7 mn	168.5 mn	41.6 no
		N180	207.6 g	400.9 df	250.9 eh	160.0 jk	305.1 ij	79.6 l
		N240	234.7 g	426.0 d	253.0 eh	163.0 jk	321.1 gh	87.7 kl
	ST	N0	168.7 g	349.3 hi	184.4 ik	107.9 n	167.1 n	31.9 op
		N120	229.8 g	454.4 c	220.7 fj	134.6 l	183.5 m	52.8 m
		N180	243.1 g	462.3 c	256.1 eg	170.9 j	327.8 fh	93.2 jk
		N240	273.0 g	470.1 c	260.1 ef	187.4 i	343.1 f	101.5 ij
I			8274.6 **	301.2 **	1859.4 **	63598.9 **	6872.2 **	16465.0 **
T			529.2 **	318.7 **	6.3 **	549.6 **	124.7 **	284.4 **
N			16.8 **	302.6 **	66.9 **	769.0 **	2788.1 **	8329.5 **
I × T			466.6 **	34.4 **	0.3 ns	176.4 **	27.9 **	164.9 **
I × N			4.9 ns	7.6 **	8.5 **	11.0 **	119.2 **	4087.0 **
T × N			1.6 ns	6.1 ns	0.5 ns	25.7 **	8.5 **	20.8 **
I × T × N			1.3 ns	0.1 ns	0.2 ns	77.4 **	8.8 **	14.6 **

I0, no-irrigation; I1, one-off irrigation; RT, PT, and ST refer to rotary tillage, plowing, and subsoiling, respectively; N0, N120, N180, and N240 refer to N managements with the N rates of 0, 120, 180, and 240 kg N ha^−1^, respectively. Different lowercase letters within columns indicate significant differences by Duncan’s test at *p* < 0.05, and only the first and last letters are retained when there are more than two lowercase letters. **, significant at *p* < 0.01; ns, not significant at *p* < 0.05.

**Table 7:** Effects of irrigation practices, tillage methods, and N management on SS content (mg g^−1^ FW) in wheat leaves during different stages in 2020–2022.

Treatment			2020–2021			2021–2022		
			Anthesis Stage	Middle Filling Stage	Later Filling Stage	Anthesis Stage	Middle Filling Stage	Later Filling Stage
I0	RT	N0	47.9 de	49.8 eh	21.4 l	38.2 fh	58.6 f	45.0 g
		N120	55.0 c	54.2 cf	24.2 ik	41.7 de	68.0 cd	52.1 ef
		N180	57.6 ac	59 ad	25.5 ij	55.3 b	83.2 b	64.2 bc
		N240	56.2 bc	58.1 bd	26.2 hi	52.2 c	80.3 b	61.4 c
	PT	N0	49.7 d	56.8 ce	23.0 jl	34.8 jk	56.8 fg	43.9 g
		N120	55.7 bc	57.1 ce	31.1 df	40.6 df	67.0 ce	51.0 ef
		N180	57.5 ac	61.1 ac	34.2 bc	55.7 b	70.4 c	62.8 c
		N240	54.9 c	61.1 ac	29.8 df	50.0 c	69.3 cd	56.1 d
	ST	N0	50.4 d	57.2 ce	26.6 gi	39.8 eg	68.9 cd	53.0 e
		N120	56.4 bc	58.8 ad	32.2 cd	42.9 d	82.9 b	53.6 de
		N180	59.5 ab	65.7 ab	36.5 ab	63.8 a	90.7 a	66.1 ab
		N240	61.6 a	66.4 a	38.6 a	64.9 a	93.8 a	68.8 a
I1	RT	N0	30.5 k	30.9 l	18.7 m	28.5 mn	48.9 i	32.2 k
		N120	32.5 jk	31.4 l	20.8 lm	33.6 k	53.8 gh	35.3 j
		N180	36.4 hj	40.0 ik	21.2 lm	33.6 kl	57.1 fg	38.8 hi
		N240	36.8 hi	40.1 ik	21.7 kl	37.6 ghi	59.1 f	38.8 hi
	PT	N0	31.9 k	33.7 kl	24.6 ij	27.4 n	47.6 i	34.7 jk
		N120	34.0 ik	36.5 jl	28.9 fg	30.9 lm	51.1 hi	35.5 j
		N180	38.9 gh	43.9 hj	29.5 ef	36.4 hj	63.3 e	39.0 hi
		N240	37.6 hi	45.4 gi	28.7 fh	37.5 gj	66.3 de	39.2 h
	ST	N0	33.9 ik	43.7 hj	20.6 lm	33.0 kl	50.9 hi	35.5 j
		N120	37.4 hi	47.6 fi	31.7 ce	35.1 ik	55.6 fg	36.2 ij
		N180	42.1 fg	47.9 fi	32.1 ce	39.6 eg	67.0 ce	44.8 g
		N240	44.3 ef	52.4 dg	33.8 c	39.6 eg	67.6 cd	49.7 f
I			1650.3 **	193.0 **	74.6 *	2584.8 **	1578.5 **	3276.8 **
T			17.2 **	20.4 **	223.0 **	102.9 **	186.3 **	203.0 **
N			46.1 **	22.5 **	120.2 **	347.1 **	262.6 **	216.7 **
I × T			1.5 ns	2.1 ns	4.3 ns	10.4 **	82.8 **	14.0 **
I × N			2.3 ns	0.5 ns	3.0 **	84.0 **	9.8 **	29.1 **
T × N			1.6 ns	0.3 ns	15.7 **	6.8 **	2.1 ns	10.3 **
I × T × N			0.2 ns	1.0 ns	4.0 **	8.4 **	11.2 **	3.4 **

I0, no-irrigation; I1, one-off irrigation; RT, PT, and ST refer to rotary tillage, plowing, and subsoiling, respectively; N0, N120, N180, and N240 refer to N management of 0, 120, 180, and 240 kg N ha^−1^, respectively. Different lowercase letters within columns indicate significant differences by Duncan’s test at *p* < 0.05, and only the first and last letters are retained when there are more than two lowercase letters *, significant at *p* < 0.05; **, significant at *p* < 0.01; ns, not significant at *p* < 0.05.

**Table 8:** Effects of irrigation practices, tillage methods, and N management on Pn (μmol CO_2_ m^−2^ min^−1^) in wheat leaves at different growth stages in 2020–2022.

Treatment	2020–2021			2021–2022		
	Anthesis Stage	Middle Filling Stage	Later Filling Stage	Anthesis Stage	Middle Filling Stage	Later Filling Stage
I0	13.0 b	11.3 b	5.2 b	13.0 b	11.8 b	3.1 b
I1	21.3 a	14.4 a	7.5 a	15.5 a	13.7 a	5.7 a
RT	16.3 b	12.3 b	5.5 c	12.9 c	12.3 b	3.7 c
PT	17.0 b	12.6 b	6.4 b	14.1 b	12.5 b	4.3 b
ST	18.2 a	13.7 a	7.2 a	15.7 a	13.4 a	5.2 a
N0	15.8 c	11.8 c	4.8 c	12.5 c	11.3 c	3.8 c
N120	16.7 b	12.7 b	6.0 b	13.8 b	12.8 b	4.3 b
N180	18.1 a	13.4 a	7.3 a	15.1 a	13.3 a	4.7 a
N240	18.0 a	13.5 a	7.4 a	15.5 a	13.5 a	4.8 a
I	254.8 **	1051.0 **	61.0 **	126.1 **	128.8 **	1067.3 **
T	21.3 **	6.5 *	36.1 **	219.0 **	8.1 **	237.5 **
N	20.4 **	14.1 **	67.3 **	34.0 **	14.3 **	33.9 **
I × T	1.4 ns	0.0 ns	0.5 ns	33.4 **	0.8 ns	52.6 **
I × N	0.9 ns	1.6 ns	3.6 *	1.8 ns	1.0 ns	2.5 ns
T × N	0.7 ns	1.0 ns	3.5 *	0.8 ns	0.4 ns	2.5 *
I × T × N	0.2 ns	0.5 ns	0.5 ns	0.4 ns	0.4 ns	3.3 *

I0, no-irrigation; I1, one-off irrigation; RT, PT, and ST refer to rotary tillage, plowing, and subsoiling, respectively; N0, N120, N180, and N240 refer to N managements with the N rates of 0, 120, 180, and 240 kg N ha^−1^, respectively. Different lowercase letters within columns indicate significant differences by Duncan’s test at *p* < 0.05, and only the first and last letters are retained when there are more than two lowercase letters *, significant at *p* < 0.05; **, significant at *p* < 0.01; ns, not significant at *p* < 0.05.

## Data Availability

Data are available within this article.
